# HHV-8 genotypes and clinical features in Kaposi sarcoma and primary effusion lymphoma in people living with HIV in Salvador, Brazil

**DOI:** 10.3389/fmicb.2026.1883222

**Published:** 2026-06-19

**Authors:** Carlos Frederico Lopes Benevides, Andreas Stocker, Sueli Moreno Vieira, Estela Luz, Carlos Brites

**Affiliations:** 1 Laboratório de Pesquisa em Infectologia, Hospital Universitário Professor Edgard Santos, Salvador, Brazil; 2 Fundação Bahiana de Infectologia, Salvador, Brazil; 3 Centro de Formação em Biologia Molecular Charles Mérieux (CFBMCM), Salvador, Brazil

**Keywords:** Brazil, genotype, HHV-8, Herpesvirus 8, Kaposi sarcoma, phylogenetic analysis, primary effusion lymphoma

## Abstract

**Introduction:**

Human herpesvirus 8 (HHV-8) is the etiologic agent of Kaposi sarcoma (KS) and primary effusion lymphoma (PEL), important causes of morbidity and mortality among people living with HIV (PLWH). The highly polymorphic ORF-K1 region is commonly used for HHV-8 genotyping and phylogenetic analysis. We investigated the distribution of HHV-8 genotypes and their clinical correlates among PLWH diagnosed with KS or PEL in Salvador, Brazil.

**Methods:**

Between 2022 and 2025, biological specimens from PLWH with KS or PEL were prospectively collected, including skin biopsies, blood, pleural effusions, and serum samples. HHV-8 DNA was detected by real-time PCR, and positive samples underwent semi-nested PCR amplification and sequencing of the ORF-K1 region. Phylogenetic analyses were performed using Maximum Likelihood reconstruction with 602 reference HHV-8 K1 sequences obtained from GenBank. Clinical, virological, and immunological data were analyzed according to HHV-8 genotype.

**Results:**

HHV-8 DNA was successfully sequenced in 14 of 19 samples. Phylogenetic analysis identified subtype C in 7/14 (50.0%) cases, subtype B in 4/14 (28.6%), and subtype A in 3/14 (21.4%). Most participants were men who have sex with men (86.6%), with a median age of 34 years. Among 13 patients with available clinical data, eight (61.5%) presented with high-risk KS or PEL. Genotype A was detected in patients with aggressive disease manifestations, including visceral KS and PEL, whereas subtype C was predominantly associated with mucocutaneous disease. However, no statistically significant association was observed between genotype and disease severity (Fisher-Freeman-Halton exact test, *p* = 0.4639). Phylogenetic reconstruction also identified a distinct cluster containing two previously reported A5 isolates that segregated from recognized HHV-8 subtypes.

**Discussion:**

HHV-8 subtype C was the predominant genotype among PLWH with KS or PEL in Salvador, followed by subtypes B and A. Although limited by sample size, subtype A was observed only in patients with severe diseases, consistent with previous reports suggesting an association between subtype A and severe clinical presentations. The identification of a distinct phylogenetic branch expands current knowledge of HHV-8 genetic diversity and warrants further investigation. These findings improve understanding of HHV-8 molecular epidemiology and provide insights for future studies evaluating genotype-phenotype associations.

## 1 Introduction

Kaposi sarcoma (KS) is a mesenchymal tumor linked to human herpesvirus 8 (HHV8) and is classified as an AIDS-defining illness. In 1989, a study from San Fracisco/USA evaluated the seroprevalence of HHV8 in 400 men with HIV and 400 seronegative controls. HHV8 antibodies were found in 223 of 593 men (37.6 percent) who reported sex with men in the previous 5 years and in none of 195 men who did not report it. Among the men with both HIV and HHV-8 at baseline, the 10-year probability of Kaposi’s sarcoma was 49.6 percent (Martin et al., 1998).

KS lesions are characterized by increased levels of proinflammatory cytokines and growth factors, which may regulate HHV-8 replication and contribute to the disease’s pathogenesis (Dorak et al., 2005; Zhang et al., 2023). In addition to Kaposi sarcoma, HHV-8-positive primary effusion lymphoma (PEL) is a newly identified type of B-cell non-Hodgkin lymphoma, characterized by liquid growth in serum body cavities. Aside from viral infection, no genetic alterations have been linked to PEL, and no consistent cytogenetic abnormalities have been found. However, the consistent monoclonality of PEL suggests that the disease is not solely driven by viral proliferation (Pérez and Tous, 2017; Gaidano et al., 1999; Fung et al., 2017; Chadburn et al., 2004; Drexler et al., 1998; Kanno et al., 2010).

Herpesvirus 8 (HHV-8), a DNA virus with a genome approximately 160–170 kb in length, encodes at least 87 open reading frames (ORFs) (Russo et al., 1996). While the overall genome is highly conserved, notable variability has been observed at both termini. At the left end of the HHV-8 genome, ORF-K1 encodes a highly polymorphic membrane glycoprotein comprising 289 amino acids. The amino acid sequence of ORF-K1 exhibits variability ranging from 0.4 to 44%, primarily concentrated within two hypervariable regions, VR1 and VR2. Several HHV-8 genotypes, including K1 subtypes A, B, C, D, E, and F, have been characterized. These subtypes may differ in pathogenicity and clinical outcome, with some evidence suggesting that subtype B is associated with a more favorable prognosis (de Oliveira et al., 2020; Kanno et al., 2010; de Lopes et al., 2021; Kouri et al., 2007; Kakoola et al., 2001).

Recently, we observed an increasing number of cases of aggressive Kaposi sarcoma characterized by extensive cutaneous and visceral lesions, even among people with HIV (PWH) on stable ART with undetectable HIV-1 viral load, in Salvador, Brazil. This study aims to investigate the frequency of HHV-8 subtypes among PWH in Salvador, Brazil, through the genetic characterization of the highly variable ORF-K1 region. Phylogenetic analysis, combined with clinical and demographic data, was employed to elucidate potential associations between HHV-8 genetic variability and disease manifestation.

## 2 Materials and methods

### 2.1 Procedures

This study included samples from PWH suspected of having KS. DNA from whole blood samples was extracted using the Purelink Genomic DNA Mini Kit (Invitrogen). DNA from tissue (biopsy) and FFPE samples, post-deparaffinization with Citrisolve (Fisher Scientific), was extracted using the Recover All Nucleic Acid Kit (Ambion/Invitrogen). The extracted materials were subsequently frozen at −80 °C (Bezold et al., 2001).

**Figure 1:**
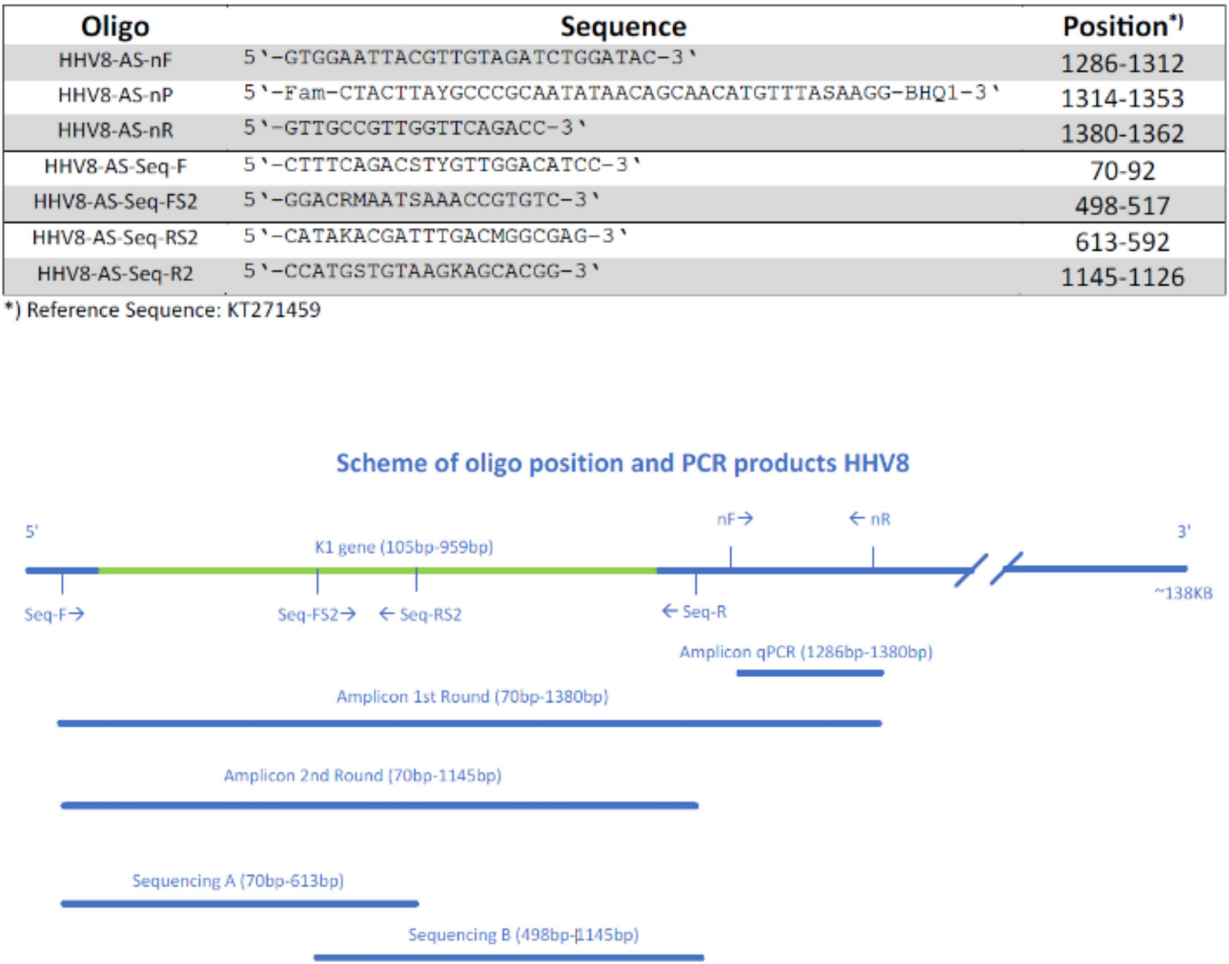
Oligo DNA and sequence rounds products.

Each sample was tested via real-time qPCR with a Taqman probe. Samples with a Ct value up to 35 were further amplified by semi-nested PCR. Primers and probes were designed based on a primary alignment of 2092 GenBank sequences, obtained using ACNUC (Lyon University) with the keywords “HHV8” (4,292 hits) and “K1” (2,902 hits). The first 1,500 bp of these sequences were aligned using Clustal Omega (64 bits, manually integrated into Seaview 64 bits, Windows GUI) and revised. Oligonucleotides, designed based on this alignment, were individually tested in BLAST (NCBI) and modified as necessary in relation to sequence frequency (Figure 1).

Real-time PCR was performed using Platinum Taq reagents (Thermo-Fisher) in a 25 μL reaction on a QuantStudio3 System (Applied Biosystems). The final concentrations were as follows: BSA 0.04 mg/mL, 4*0.2 mM dNTPs, 4.0 mM MgCl2, 0.05 μM ROX, oligos HHV8-AS-nF 0.3 μM, HHV8-AS-nR 0.3 μM, HHV8-AS-nP 0.2 μM, Taq Polymerase 0.02 U/μl, and 5 μL eluted DNA. The volume was completed with ultra-pure PCR water. The thermal cycle began with an initial denaturation at 94 °C for 2 min, followed by 45 cycles of 20 s at 94 °C for cycle denaturation and 40 s at 60 °C for annealing/extension.

The real-time PCR assay for HHV-8 was not intended for absolute quantification but rather served as a secondary verification of the extraction procedures and nested PCR performance. Samples achieving a Ct value below 35 in the qPCR assay were considered to contain sufficient material in the second round of nested PCR for subsequent purification and sequencing. With a slope of −3.37, the PCR assay achieved an efficiency of 97.9%, with an R^2^ value of 0.998.

The first round of PCR was also performed with Platinum Taq reagents in a 50 μL reaction. The final concentrations were the same as the real-time PCR, but with oligos HHV8-AS-Seq-F 0.3 μM, HHV8-AS-nR 0.3 μM, and 5 μL eluted DNA. The thermal cycle was like the real-time PCR, but with 35 cycles and an annealing temperature of 57 °C. The second round of the semi-nested PCR was performed in a 60 μL reaction with the same reagents and concentrations, but with 1 μL of the first-round PCR product and oligos HHV8-AS-Seq-F 0.3 μM, HHV8-AS-Seq-R2 0.3 μM. The thermal cycle was the same as the first round of PCR, but with a 75-s extension at 72 °C.

The second-round PCR products were purified using the Purelink PCR Purification mini-Kit (Invitrogen) and stored at −20 °C. These products were then used in the Big Dye Terminator 3.1 reaction (Applied Biosystems) using the two primers from the second PCR round (HHV8-AS-Seq-F and HHV8-AS-Seq-R2) and two sequencing primers (HHV8-AS-Seq-FS2 and HHV8-AS-Seq-RS2), following the manufacturer’s standard protocol. The products were purified by isopropanol precipitation and sequenced using SeqStudio (Sanger technology/Applied Biosystems). The quality of the sequences was evaluated using Trev software and Pregap 4, and the sequences were aligned using Gap 4 (all parts of the Staden Package).

To obtain all relevant sequences for the phylogenetic tree, a complete K1 gene sequence (870 bp) was searched in BLAST with the blastn configuration (2095 hits). Complete GenBank files were downloaded, and the country and subtype were extracted from GenBank features. Finally, 602 complete K1 sequences (from start to stop codon, excluding deletions without frameshift) were translated into protein sequences (280–290 AA) along with our sample sequences. All sequence analysis and translation in this project were performed using BioEdit software. The amino acid alignment was tested using MEGA11’s internal test to find the best model. A phylogenetic tree was then created using the Maximum Likelihood method by the Jones-Taylor-Thornton model with Gamma Distribution and 1,000 bootstraps.

### 2.2 Ethical statement

The study was conducted according to the Declaration of Helsinki and was approved by the Institutional Board Review, (Hospital Universitário Professor Edgard Santos-HUPES, Federal University of Bahia, protocol number: 2.832.026). All information given to the research team was de-identified.

### 2.3 Data analysis

Clinical data (HIV viral load, log10 viral load, and CD4 + T-cell count) and HHV-8 genotypes were recorded. Continuous variables were summarized as mean ± SD or median (IQR), as appropriate. Normally distributed variables were compared across genotype groups using one-way ANOVA; non-normally distributed variables were compared using the Kruskal–Wallis test. Categorical variables were reported as *n* (%) and compared using Pearson’s chi-square test or Fisher’s exact test. Tumor burden was dichotomized as high (visceral or extensive skin/mucosal disease and PEL) versus low (skin-only or low mucosal disease) and compared by genotype using Fisher–Freeman–Halton (FFH) exact test; odds ratios with 95% confidence intervals were calculated from contingency tables. Statistical analyses were performed in R version 4.5.1 (R Foundation for Statistical Computing). Two-sided *p*-values <0.05 were considered statistically significant.

## 3 Results

As described in methodology, paraffin-embedded tissue biopsies were initially collected from seven patients diagnosed with KS at the Pathology Laboratory of HUPES.

We tried to extract DNA from these paraffin-embedded tissue, but no viable DNA was obtained, likely due to extensive DNA fragmentation. Notably, at the time of biopsy collection, the samples were fixed in formaldehyde rather than buffered formalin, which may have adversely affected DNA integrity and contributed to the poor extraction outcomes. From 2022 to 2025, a prospective cohort of 13 patients was enrolled for skin biopsies performed *in vivo*. Blood samples were obtained from patients without cutaneous lesions, in cases biopsy could not be performed or presented exclusively with visceral disease. Additionally, pleural effusion samples from three patients diagnosed with PEL and one serum sample were collected from the hospital’s biorepository. All samples underwent DNA extraction, with detailed results summarized in Table 1. Out of 19 samples analyzed, five yielded negative results for HHV-8 DNA — four derived from skin biopsies and one from blood. All pleural effusion and serum samples were positive for HHV-8 DNA in this study.

**Table 1:** Results from HHV-8 DNA extraction in biological specimens from patients living with HIV and diagnosed with Kaposi sarcoma or Pleural effusion lymphoma.

Sample	Patient number	Biopsy site	HHV8 – DNA extration
1	KS1	Skin	Negative
2	KS2	Skin	Positive
3	KS3	Skin	Positive
4	KS4	Skin	Positive
5	KS5	Skin	Positive
6	KS6	Skin	Positive
7	KS7	Skin	Negative
8	KS8	Skin	Positive
9	KS9	Skin	Positive
10	KS10	Blood	Negative
11	KS11	Skin	Positive
12	KS12	Skin	Positive
13	KS13	Skin	Negative
14	KS14	Pleural effusion	Positive
15	KS15	Pleural effusion	Positive
16	KS16	Pleural effusion	Positive
17	KS17	Serum	Positive
18	KS18	Skin	Negative
19	KS19	blood	Positive

### 3.1 Epidemiological and demographic data

Epidemiological data was available for 15 of the 19 patients. The median age was 34 years old. Among these, 14 were male and one female. Regarding male patients, thirteen were identified as men who have sex with men (MSM) (86.6% of 15 participants) and one reported sex with men and women. The only female patient reported a history of sex work and substance abuse prior to HIV diagnosis. Considering race and ethnicity, all patients were Hispanic (Brazilian) origin, with three declared as white and twelve as black or pardo. Most patients had completed elementary or middle school education, with only two having higher academic education. Data on HIV viral load and CD4 T-cell counts were incomplete; therefore, these parameters are presented separately. Demographic and epidemiological characteristics are summarized in Table 2.

**Table 2:** Demographic and epidemiological data from patients living with HIV and diagnosed with Kaposi sarcoma.

Sample	Patient number	Race	Sex	Sexual orientation	Educational stage
1	KS1	Multiracial (pardo)	Male	MSM	NA
2	KS2	Multiracial (pardo)	Male	MSM	Upper secondary
3	KS3	Mutliracial (pardo)	Male	Bisexual	Upper secondary
4	KS4	Black	Male	MSM	Upper secondary
5	KS5	White	Male	MSM	Upper secondary
6	KS6	Black	Male	MSM	Primary education
7	KS7	Multiracial (pardo)	Male	MSM	Primary education
8	KS8	Black	Female	Heterosexual	Primary education
9	KS9	White	Male	MSM	Upper secondary
10	KS10	White	Male	MSM	Bachelor’s academic
11	KS11	Black	Male	MSM	Upper secondary
12	KS12	Multiracial (pardo)	Male	MSM	Bachelor’s academic
13	KS13	Multiracial (pardo)	Male	MSM	Upper secondary
14	KS14	NA	NA	NA	NA
15	KS15	NA	NA	NA	NA
16	KS16	NA	NA	NA	NA
17	KS17	NA	NA	NA	NA
18	KS18	Multiracial (pardo)	Male	MSM	Primary education
19	KS19	Multiracial (pardo)	Male	MSM	Primary education

MSM, men who have sex with men; NA, not available.

### 3.2 HHV-8 phylogenetic analysis

**Figure 2:**
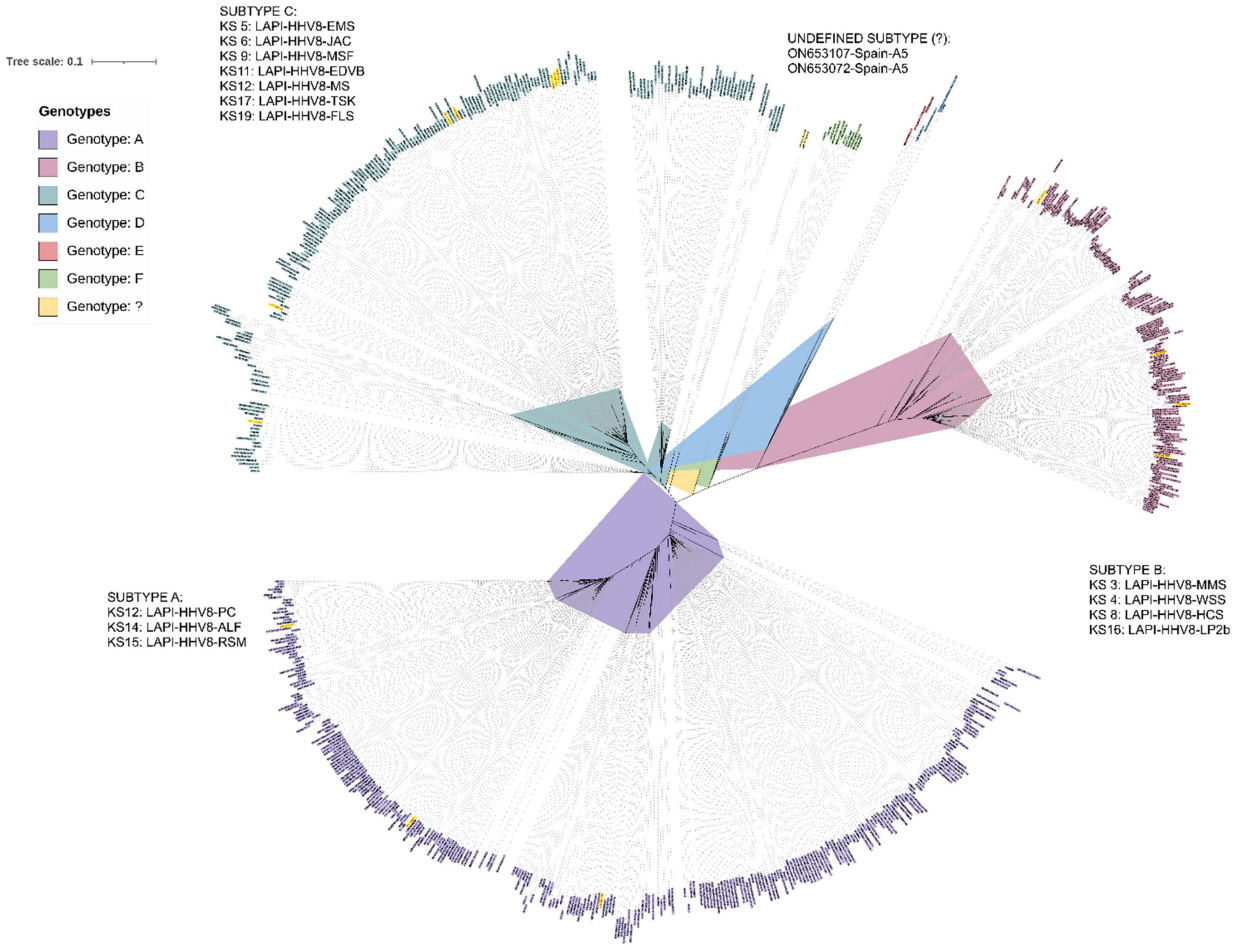
HHV-8 phylogenetic analysis hierarchical. Fourteen HHV-8 samples underwent phylogenetic analysis. Three (3) were characterized as genotype A, four (4) were characterized as genotype B and seven (7) were characterized as genotype C. The phylogenetic analysis presents two (2) samples from GenBank library, previously described as genotype A, clustering in a distinct phylogenetic branch represented in figure as undefined (?).

HHV-8 DNA was successfully extracted from 14 of the 19 collected samples. The unsuccessful extractions are likely due to low viral load, associated with regressing lesions and minimal viral replication. Sequencing was performed on these samples following established protocols, and phylogenetic analysis was conducted using BioEdit software. The resulting phylogenetic tree is shown in Figure 2.

HHV-8 genotypes were classified into subtypes A, B, and C, which were identified in 21.4, 28.6, and 50% of patients, respectively. Specifically, from 14 individuals, subtype A was detected in three, subtype B in four, and subtype C in seven. Among the three samples from patients with PEL, two harbored subtype A, and one subtype B. In patients with KS, six had subtype C, three had subtype B, and one had subtype A. The serum sample was identified as genotype C.

Clinical correlations were evaluated in 13 patients, of whom eight (61.5%) presented with high-risk KS or PEL, while five (38.5%) had low-risk disease. Genotype A was identified only among patients with severe disease, including one case of high-risk KS and two cases of PEL. These findings suggest a possible association between genotype A and more aggressive HHV-8-related disease manifestations, such as visceral involvement, extensive mucocutaneous disease, and PEL. However, no statistically significant association was observed between HHV-8 genotype (A, B, or C) and disease severity (Fisher–Freeman–Halton exact test, *p* = 0.4639). The lack of statistical significance is likely attributable to the limited sample size (*N* = 13). Conversely, subtype B was predominantly identified in patients with a high burden of cutaneous lesions, but no visceral involvement. In our cohort, all patients presenting with exclusively mucosal lesions or limited skin lesions were associated with subtype C. Clinical data was unavailable for one serum sample related to subtype C. No correlation was observed between HIV viral load, CD4 T-cell counts, and clinical presentation at diagnosis (*p*-value: 0.8383, Kruskal-Wallis test); however, patients infected with subtype C tended to exhibit lower viral loads and/or higher CD4 counts. Demographic and clinical characteristics, along with HHV-8 subtypes, are summarized in Table 3.

**Table 3:** Clinical data from patients living with HIV and diagnosed with Kaposi sarcoma and HHV-8 genotype.

Sample	Patient number	Site of disease	HIV (copies/mL)	CD4 cell count	HHV8 - Subtype
1	KS1	Skin (low burden)	0	584	Negative
2	KS2	Skin, mucosal and visceral	527,894	35	A
3	KS3	Skin (high burden), penis	70,100	42	B
4	KS4	Skin (high burden, oedema)	21	139	B
5	KS5	Skin (high burden), mucosal	58	80	C
6	KS6	Skin (high burden)	23	346	C
7	KS7	Skin (low burden)	233	411	Negative
8	KS8	Skin (high burden, oedema)	0	1,007	B
9	KS9	Skin (Low burden)	389,171	50	C
10	KS10	Skin (low burden), mucosal	0	548	Negative
11	KS11	Skin (low burden, mucosal)	118	585	C
12	KS12	Skin (low burden)	NA	NA	C
13	KS13	Skin (low burden)	960,000	11	Negative
14	KS14	Pleural effusion	NA	NA	A
15	KS15	Pleural effusion	NA	NA	A
16	KS16	Pleural effusion	NA	NA	B
17	KS17	Unknown	NA	NA	C
18	KS18	Skin (low burden)	0	1,000	Negative
19	KS19	Skin, mucosal and visceral	598,000	5	C

HIV, Human Immunodeficiency Virus; CD4, CD4 + lymphocyte count; HHV-8, Herpes virus 8; NA, not available.

### 3.3 HHV-8 subtypes review after phylogenetic analysis

In the final analysis of HHV-8 phylogenetic tree, we observed that two Genbank cases, previously described from Spain as subtype A5, clustered in a distinct phylogenetic branch, that diverge from subtypes A, B, C, D, E, and F, and we designated here as undefined genotype (Figure 2, Supplementary Figure 1). Reviewing published data from geographic distribution in the phylogenetic characterization, was observed that subtype A was predominantly found in Europe, with additional presence in Malawi and Uganda (Sub-Saharan Africa). Subtype B was mainly reported in the United States, Brazil, Uganda, and Zambia (Sub-Saharan Africa). Subtype C was primarily detected in Spain, Greece, and Morocco (North Africa), as illustrated in Supplementary Figure 1.

## 4 Discussion

We performed HHV-8 genotyping by characterizing the K1 gene region. Early reports on HHV-8 genotyping revealed that the K1 region is more variable than other regions of the virus (Meng et al., 1999). Our findings indicate that HHV-8 subtype C is the most prevalent among patients in Bahia, accounting for 50% of cases, followed by subtypes B (28.6%) and A (21.4%).

These results partially contrast with earlier epidemiological studies of KS. In Brazil, a previous study analyzed 50 KS cases and reported that the most frequently detected HHV-8 genotypes were A (50.0%) and C (48.0%), with genotype B identified in only one case (2.0%). Moreover, subtype A was predominantly associated with KS lesions in PWH, whereas subtype C was more common among individuals without HIV (Ramos da Silva et al., 2011). Similarly, a study in Argentina, reported HHV-8 detection in 50.7% of samples from PWH, 59.2% from KS patients, and 8% from blood donors. Phylogenetic analysis revealed the presence of subtypes A (46.9%), B (6.25%), C (43.75%), and F (3.1%) within the sampled population (Hulaniuk et al., 2020).

A recent study describing the global distribution of HHV-8 indicated that genotypes A and C have similar worldwide prevalence, predominantly occurring in Africa and Europe. Genotype B was most common in Africa. Among the rare genotypes, subtype D was reported in East Asia and Oceania, E in South America, and F was also primarily found in Africa. The highest genotypic diversity was observed across the Americas, with Brazil harboring five HHV-8 genotypes—A (31.1%), B (32.8%), C (31.1%), E (3.3%), and F (1.6%) (de Lopes et al., 2021). This distribution reveals nearly equal prevalences of subtypes A, B, and C, contrasting with our findings, where subtype C was the most prevalent, accounting for 50% of cases, followed by subtype B at 28%. Our data align with a more recent Brazilian study by de Oliveira Lopes et al., which analyzed HHV-8 DNA from 24 positive samples from individuals with HIV/AIDS and KS in São Paulo and Rio de Janeiro. Phylogenetic analysis revealed that genotypes C, A, and B were present in 45.8, 29.2, and 25% of the isolates from Brazil, respectively, further supporting the regional distribution patterns observed in our study (de Oliveira et al., 2020).

According to the phylogenetic analysis, HHV-8 subtype B isolates appear to originate from regions in Sub-Saharan Africa, including Uganda, Zambia, and Congo. In contrast, subtypes A and C seem to have originated from Europe—specifically Spain and Russia—and North Africa, such as Morocco. These findings are partially consistent with those reported by Oliveira et al., who noted that subtype A isolates derive from Ukraine, Russia, and the Tatar ethnic group; subtype B isolates from Congo and the Democratic Republic of Congo; and subtype C isolates from regions including Australia, Algeria, England, and French Guiana (de Oliveira et al., 2020; Yogi et al., 2025; Mohanna et al., 2005; Zong et al., 1999; Beyari et al., 2004; Tozetto-Mendoza et al., 2016; Gómez et al., 2022).

We propose that the increasing prevalence of HHV-8 subtype B in Bahia and throughout Brazil may be partly attributable to the demographic characteristics of the Brazilian population. Brazil is a multiethnic country, with Bahia historically serving as a major hub for European and, predominantly, African immigration since early colonization. Approximately 50% of the Brazilian population and 80% of Bahia’s population are of African descent, which could influence the distribution of HHV-8 subtypes, particularly subtype B.

A systematic review on seroprevalence data among African populations reported a seroprevalence range from 2.0% in young children in Eritrea to 100% in individuals with KS in the Central African Republic, as well as in a broader cohort with KS in Morocco. Data on K1 genotypes were available for approximately 38% of the countries studied. The reported frequencies of K1 genotypes showed a higher subtype B prevalence (42.1%), compared to A (5.3%), A5 (26.3%), B-C (18.4%), F (5.3%), and Z (2.6%) subtypes (Etta et al., 2018).

In the present study, clinical correlations indicated that subtype A was associated with more aggressive KS manifestations, including visceral involvement, extensive mucocutaneous disease, and PEL, a severe variant of non-Hodgkin lymphoma. Subtype B was predominantly found in subjects with cutaneous lesions, with high burden disease in our scenario, but with no visceral or mucosal involvement. Most patients with subtype C were associated with mucosal involvement, skin lesions and only one of them with visceral involvement, harboring high HIV viral loads and very low CD5 (Brown et al., 2006; Kumar et al., 2007).

The association of subtype A with more aggressive forms of the disease is consistent with existing literature (Yanik et al., 2016; Cesarman et al., 2022). In 1998, Boralevi et al. identified distinct epidemiological patterns of HHV-8 in 43 patients from France and the United States. Subtype C was rare among American and European KS patients but was predominant in Italian patients with sarcoidosis. The study also observed some differences in the clinical evolution of KS and HIV infection based on HHV-8 subtypes. Patients with subtype A exhibited a significantly higher occurrence of mucosal and/or visceral lesions (62.5% for subtype A versus 27% for subtype B), suggesting that subtype A, which was found predominant in the United States, may have more aggressive pathogenic characteristics (Boralevi et al., 1998).

Regarding subtypes B and C, a 2016 study by Tozetto-Mendoza et al. analyzed saliva and blood samples from 37 PWH with active KS. The distribution of HHV-8 genotypes was 25% for subtype A, 37.5% for subtype B, and 37.5% for subtype C. The clinical data suggested a potential correlation between subtype B and a better prognosis, as patients predominantly presented with smaller, localized cutaneous lesions compared to other subtypes (Tozetto-Mendoza et al., 2016). However, in this study, all subtypes included patients with visceral or disseminated disease, contrasting with our findings, where only subtypes A and C manifested with visceral or mucosal involvement.

Despite the small sample size (*n* = 7), we hypothesize that HHV-8 subtype C may be associated with predominantly mucosal and cutaneous manifestations in participants on stable ART. The patients in our cohort exhibited lower HIV viral loads and/or higher CD4 cell counts, suggesting a preserved immune status. Consequently, we propose that these patients may have a clinical course like classical KS. Supporting this, a recent study by Yogi et al. examined the relationship between HHV-8 genotypes and clinical presentation in KS patients from Okinawa, Japan, an endemic region. Their findings indicated that genotypes A and C were detected in 94% of patients with classic KS, with 72% infected with single-genotype C. Conversely, patients with AIDS-related KS predominantly harbored genotype A infections (80%), suggesting potential differences in pathogenicity and clinical behavior among genotypes (Yogi et al., 2025; Beral et al., 1990; Joo et al., 2006).

HHV-8 is an evolving virus. A pivotal study by Zong et al. proposed that the global distribution of the three major HHV-8 subtypes (A/C, B, and D) aligns closely with patterns of familial transmission and supports the continental host migration model, which posits that modern humans originated as a distinct species in East Africa approximately 150,000 years ago. This evolutionary analysis estimates that genotype B emerged around 100,000 years ago within Africa, while genotypes D and E appeared approximately 60,000 years ago in the Pacific Islands and among Native American populations, respectively. Additionally, genotypes A and C are believed to have arisen roughly 35,000 years ago in Eurasia. These findings underscore the dynamic evolutionary history of HHV-8 and its co-migration with human populations (Zong et al., 2002; Ishak et al., 2007).

Surprisingly, our phylogenetic analysis revealed a distinct genotypic branch, which we designate as undefined genotype. This subtype was identified in two HHV-8 subjects, both previously classified as subtype A5 in a 2022 study by Gómez et al. In that investigation, samples from 142 HHV-8-infected patients in Spain were analyzed to assess viral subtype diversity. Most KS patients harbored subtypes C3 (13 patients, 27.1%) and A3 (13 patients, 27.1%), followed by subtype A5, identified in 8 patients (16.7%). Regarding KS classifications, 3 cases (6.25%) were identified as endemic KS with subtype A5; 8 cases (16.6%) as epidemic KS with subtype A3; 7 cases (14.6%) as classical KS; and 2 cases (50%) as iatrogenic KS, both associated with subtype C3 (Gómez et al., 2022).

We hypothesize that these subtypes could represent an evolutionary divergence from the ancestral B genotype, rather than a derivative subtype A, as previously suggested by Gómez et al. In their study, subtype A5 was present in 20 patients (14.1%), with 8 attributed to African origins based on endemic KS cases from Nigeria, Cameroon, Equatorial Guinea, and Morocco. This intriguing finding warrants further investigation and analysis of additional samples to confirm the existence and significance of this novel genotype.

This study has some limitations, like the relatively small sample. Challenges with DNA extraction from archived formaldehyde-fixed tissue, as well as incomplete data on HIV viral load and CD4 counts, limited the number of samples that could be analyzed. Our cross-sectional study design limits our ability to make causal or temporal inferences regarding viral dynamics. On the other hand, existing data on clinical features and HHV-8 genotypes is limited, and our study contributes significantly by enhancing the understanding of HHV-8 diversity and its clinical associations in Salvador, Brazil. The prospective collection of clinical specimens, with matched epidemiological data, utilized a K1-based genotyping and phylogenetic analysis through a reproducible pipeline. Including PEL cases further broadens the clinical spectrum addressed. The identification of a distinct phylogenetic branch, not reported previously, offers an intriguing platform for exploratory genotype–phenotype analysis. These aspects support hypothesis generation and lay a foundation for future larger, multicenter, longitudinal studies incorporating whole genome sequencing and more comprehensive clinical datasets to validate and expand upon our findings.

## 5 Conclusion

Our study shed light on the association between HHV-8 genotypes and distinct clinical and epidemiological patterns in Bahia, Brazil. The predominance of subtype C aligns with prior regional reports, while the presence of subtypes B and A suggests complex transmission dynamics influenced by demographic factors, including ancestry and sexual behavior. Consistent with global and Brazilian data, subtype A was linked to more aggressive disease phenotypes, such as visceral involvement and primary effusion lymphoma, whereas subtype C appeared associated with a less aggressive, mucosal, and cutaneous presentation in individuals with better immune status under ART. These findings correlated with detection of a distinct genotype, possibly highlight the ongoing viral evolution and underscore the importance of continuous genotyping surveillance. Overall, our findings contribute to the understanding of HHV-8 genetic diversity and its role in KS pathogenesis, emphasizing the need for larger, multicenter studies to elucidate the clinical implications of viral genotypes and to inform tailored management strategies for affected populations.

## Data Availability

The datasets presented in this study can be found in online repositories. The names of the repository/repositories and accession number(s) can be found in the article/Supplementary material.

## Supplementary material

SUPPLEMENTARY FIGURE S1 HHV-8 phylogenetic analysis hierarchical. Fourteen HHV-8 samples underwent phylogenetic analysis. Three (3) were characterized as genotype A, four (4) were characterized as genotype B and seven (7) were characterized as genotype C. The phylogenetic analysis presents two (2) samples from GenBank library, previously described as genotype A, clustering in a distinct phylogenetic branch represented in figure as undefined (?).
